# Foliar application of alpha-ketoglutarate plus nitrogen improves drought resistance in soybean (*Glycine max* L. *Merr.*)

**DOI:** 10.1038/s41598-022-18660-4

**Published:** 2022-08-24

**Authors:** Zhijia Gai, Jingqi Liu, Lijun Cai, Jingtao Zhang, Lei Liu

**Affiliations:** 1 Jiamusi Branch, Heilongjiang Academy of Agricultural Sciences, Jiamusi, 154007 Heilongjiang Province China; 2 College of Resources and Environment, Jilin Agricultural University, Changchun, 130102 Jilin Province China; 3 Northeast Institute of Geography and Agroecology, Chinese Academy of Sciences, Changchun, 130102 Jilin Province China

**Keywords:** Biochemistry, Physiology, Plant sciences

## Abstract

The aim of the present research was to understand the impacts of foliar nitrogen and α-oxoglutarate on proline accumulation, photosynthesis, and ammonium assimilation of soybean seedlings subjected to drought stress. The data in the present study demonstrated that foliar α-oxoglutarate and nitrogen significantly enhanced leaf glutamine synthetase (GS) activity, glutamate dehydrogenase (GDH) activity, glutamate content, proline content, relative water content (RWC) and photosynthesis of soybean seedlings exposed to drought stress at each stage. Accordingly, the ammonium content was significantly reduced by foliar α-oxoglutarate and nitrogen. These results suggested that a combination of foliar nitrogen plus α-oxoglutarate had an advantage over either foliar nitrogen or foliar α-oxoglutarate in increasing the proline accumulation under drought stress and a combination of foliar nitrogen plus α-oxoglutarate could better mitigate the adverse impacts of drought stress.

## Introduction

In recent years, the influence of frequent drought hazards on crop production has been increasing in Northeast China. Soybean (*Glycine max* (L.) Merr.) is the second most important crop in Northeast China (following corn), and frequently subjected to drought stress (DS) during its growth and development, which results in a significant reduction in soybean seed yield. Soybean is a vital crop which needs a sufficient supply of water during its growth process to achieve high seed yield^1^. Plants have evolved many different strategies to cope with abiotic stress. Osmoregulation is a vital mechanism for plants to acclimate to adverse osmotic stresses^1^. Proline (Pro) is reported to act as a key osmolyte in increasing the tolerance of plants^2^. Pro accumulation has been revealed to take place after drought stress in plants^3^. Drought stress leads to a wide range of physiological changes, such as stomata to close and cells to lose water. These changes disturb regular nitrogen metabolism, carbon metabolism, and result in plant death as a consequence of the decrease in relative water content (RWC). The RWC is an important parameter of plant water status^4–6^. Drought stress causes the decrease in photosynthesis rate, and alters carbon metabolism in plant, leading to depleted energy and reduced yield^7^.

Nitrogen (N) metabolism is one of the most basic metabolic processes, which affects the growth state, yield and stress tolerance of plants. There has been report on the association between proline accumulation and the acquisition of stress tolerance^8^. It is a fact that proline is eventually synthesized from glutamate (Glu) that acts an important role in the amino acid metabolism of plant^9,10^. Glu biosynthesis is much associated with ammonium uptake^10^. Excessive ammonium leads to the toxic impacts on plants when subjected to adverse abiotic stresses^11^. The GDH pathway is considered as a complementary route when plants are exposed to abiotic stresses^12,13^. Reducing the accumulation of NH_4_^+^ in plant tissues is considered to be an important ability to resist drought stress. Glutamine synthetase (GS) and glutamate dehydrogenase (GDH) are key rate limiting enzymes in the process of NH_4_^+^ assimilation and plants use NH_4_^+^ to catalyze the biosynthesis of Glu^14,15^. Nitrogen (N) is required by plants in relatively large amounts, unlike the other plant nutrients. In addition to supplying a nutrient for plant growth, nitrogen application could increase the drought tolerance of plant to enhance yield when exposed to drought stress^6,16^. Adequate nitrogen supply can enhance the drought tolerance of plants^16,17^. Yang et al.^18^ reported that nitrogen application to wheat (*Triticum aestivum* L.) during grain filling enhanced the remobilization of stored carbohydrates from vegetative plant part to grain under moderate drought stress. Several studies have provided the evidence of the role of nitrogen in ameliorating the effects of drought stress by enhancing proline accumulation, glycine betaine, and soluble protein^18–21^. Foliar application of nitrogen much more raised the RWC and the nitrate reductase activity in maize under short-term drought stress^22^.

α-oxoglutarate, an important organic acid of the tricarboxylic acid (TCA) cycle^23,24^, participates in a wide array of reactions in distinct plant cell compartments^25^, also being a key metabolite at the crossroads of carbon/nitrogen (C/N) metabolism as it is needed for assimilating ammonia^26,27^. There are some reports on the impacts of exogenous α-oxoglutarate on nitrogen assimilation in crops, such as wheat^28^, tobacco^29,30^, and rice^31^ when exposed to abiotic stress. There is a report^32^ indicating that exogenous application of α-oxoglutarate sharply reduced the NH_4_^+^ concentrations and increased the ammonium assimilation in tomato roots and shoots as compared to control (CK). The previous study indicated that exogenous α-oxoglutarate significantly enhanced the cold resistance by increasing leaf ammonium assimilation, proline accumulation and photosynthesis of soybean seedling when subjected to cold stress^33^.

However, the effects of foliar nitrogen or α-oxoglutarate, particularly the combination of foliar nitrogen plus α-oxoglutarate and the underlying mechanism were not reported in crops exposed to drought stress. This study is the first report on the effects of foliar nitrogen plus α-oxoglutarate on soybean subjected to abiotic stress. We hypothesize that: (1) the combination of foliar nitrogen plus α-oxoglutarate could improve the drought tolerance of soybean; (2) exogenous α-oxoglutarate or nitrogen helps to enhance nitrogen metabolism under drought stress.

## Materials and methods

### Plant materials and growth conditions

A pot experiment was conducted in the rain shelter at the experimental station of Northeast Agricultural University, China. The experimental pot diameter was 16 cm, and the depth was 18 cm. Each pot was filled with 600 g of typical black soil. Two spring soybean varieties, Henong51 (drought resistant) and Henong43 (drought sensitive), were used in the present study and the experiment has adopted Randomized Block Design. Henong51 was released in 2006 with 100-seed weight of 19.0–20.0 g and the plant height was 80–85 cm. The content of its protein and oil was 40.15% and 21.31%, respectively. The effective accumulative temperature for maturity (≥ 10 °C) was about 2200 °C. Henong43 was released in 2002. The 100-seed weight was 20.0–22.0 g and the plant height was 90–100 cm. The content of its protein and oil was 42.05% and 20.52%, respectively. The growth habit of the two soybean varieties was semi-determinate with purple flowers.

Soil was collected at a depth of 20 cm from a corn field. The soil was analyzed in Sangjiang River Key Laboratory of Cultivation and Breeding of Main Crops. The properties of soil were as follows: total nitrogen 2.19 g/kg; available nitrogen 139.21 mg/kg; total phosphorus 1.72 g/kg; available phosphorus 37.25 mg/kg; total potassium 27.03 g/kg; available potassium 123.25 mg/kg; organic matter 30.13 g/kg; pH 6.98.

We state that our experimental research on soybeans comply with the relevant institutional, national, and international guidelines and legislation.

### Experimental design and sampling

Twelve seeds of each soybean variety were seeded by hand in the pots. Soybean was not inoculated or dressed before planting. The main treatments included two soil water levels: (1) CK, that is, soil water content which was maintained at 19 ± 1% of water content (85 ± 5% of water-holding capacity); and (2) drought stress (DS) which was maintained at 15 ± 1% of water content (65 ± 5% of water-holding capacity). Subtreatments were (1) 0.5% Urea (N 46%), (2) 5 mmol/L α-oxoglutarate, and (3) 0.5% Urea + 5 mmol/L α-oxoglutarate. Foliar α-oxoglutarate and nitrogen were applied to soybean seedlings at V2 stage (completely unrolled leaf at the second node on the main stem). There were five treatments in the present experiment: DS1 (foliar N), DS2 (foliar α-oxoglutarate), DS3 (foliar N plus α-oxoglutarate), CK1 (foliar water under DS), CK2 (normal water content). Leaf samples for all treatments were collected after drought stress of 24 h (S1 stage), 48 h (S2 stage) and 72 h (S3 stage). By using a weighing method, the weight of each pot was recorded once every day. The amount of watering required was then calculated. When the soil RWC was lower than the lower limit of the water control treatment, water was precisely added to the pot.

We state that we have permission for foliar spraying and collecting leaves at V2 stage and there is no issues on ethics.

### Determination of nitrogen metabolism

The activity of GS and NADH-GDH was measured with the method of Lu et al.^34^. One unit of NADH-GDH activity was the reduction amount of 1 µmol of coenzyme (NADH) per min under 30 °C and one unit of GS activity was defined as the amount of enzyme catalyzing the formation of 1 µmol γ-glutamylhydroxamate per min under 37 °C.

### Determination of photosynthetic index

Photosynthetic rate was measured according to Gai et al.^35^. Photosynthetic rate was determined by using the CI-340 portable photosynthesis measuring system (CID, Inc., USA). Relative chlorophyll content (*SPAD*) in the middle leaflet of the trifoliate leaf at V2 stage was determined by SPAD-502 Chlorophyll Meter produced by Konica Minolta Inc., Japan.

### Determination of proline

Free proline was determined with the method of Bates et al.^36^. Fresh leaf (0.5 g) was used to extract proline with 3% sulphosalicylic acid, ninhydrin reagent containing glacial acetic acid and incubated under 100 °C for 1 h. The reaction mixture was rapidly cooled by using ice water. The toluene was used to extract the colored reaction product, and the toluene phase absorbance was determined at 520 nm.

### Determination of ammonium content

The ammonium content in soybean leaf was measured by using HPLC (Agilent 1100, USA) with the method of Lu et al.^35^.

### Determination of relative water content

The leaf RWC was determined through recording the turgid weight of 1.0 g fresh leaf samples by making them stay in water for 4 h, followed by drying in the hot air oven until stationary weight was derived. The following equation was used to determine RWC^37^.

$${\text{RWC (}}\% {)} = [({\text{FW}} - {\text{DW}}){/}({\text{TW}} - {\text{DW}})] \times {1}00$$

The FW is defined as sample fresh weight, TW is defined as the sample turgid weight, and DW is defined as the sample dry weight.

### Statistical analyses

Data analysis was conducted using SPSS 22 (SPSS Inc., Chicago, IL, US). Test for significance was carried out by analysis of variance, and LSD test was used to determine the significant differences (*P* < 0.05) between different treatments.

## Results and discussion

### Effects of foliar α-oxoglutarate and nitrogen on soybean fresh weight (FW)

The growth of soybean seedlings was evaluated by measuring the fresh weight. Inhibition in soybean fresh weight was significantly mitigated by foliar addition of nitrogen and α-oxoglutarate under drought stress. The data in Table 1 indicated that foliar nitrogen and α-oxoglutarate significantly enhanced the fresh weight of soybean seedling under drought stress compared to CK1 at S1, S2, and S3 stages. The fresh weight of soybean seedling under DS1, DS2, and DS3 was significantly lower than that under CK2 at each stage. There was no significant difference in fresh weight among DS1, DS2, and DS3 treatments. With the stress duration prolonged, the fresh weight of soybean seedling gradually decreased under drought stress. The fresh weight of soybean seedling under CK2 gradually increased from S1 to S3 stage, while the fresh weight of soybean seedling under DS1, DS2, DS3, and CK1 gradually decreased from S1 to S3 stage. When drought stress was applied, the fresh weight of drought-treated plants was reduced for two soybean varieties. The fresh weight of Hefeng51 was significantly lower than that for Hefeng43 at each stage. The data in the present study indicated that there was a lower reduction in fresh weight for Hefeng51 than that for Hefeng43 from S1 to S3 stage when exposed to drought stress.

**Table 1:** Effects of foliar α-oxoglutarate and nitrogen on fresh weight under drought stress (g plant^−1^).

Treatment	S1 stage		S2 stage		S3 stage	
	Hefeng51	Hefeng43	Hefeng51	Hefeng43	Hefeng51	Hefeng43
DS1	3.31^b^	3.82^b^	3.25^b^	3.69^b^	2.98^b^	3.21^b^
DS2	3.36^b^	3.86^b^	3.31^b^	3.70^b^	3.01^b^	3.32^b^
DS3	3.41^b^	3.91^b^	3.37^b^	3.72^b^	3.08^b^	3.38^b^
CK1	3.02^c^	3.59^c^	2.78^c^	3.28^c^	2.48^c^	2.71^c^
CK2	3.84^a^	4.25^a^	3.95^a^	4.31^a^	4.11^a^	4.49^a^
**Analysis of variance (ANOVA)**						
Treatment (T)	**	**	**	***	***	***
Cultivar (C)	***		***		**	
C × T	**		***		**	

Means with same letter within columns are not significantly different (*P* < 0.05) using LSD test.

***, **Significance at 0.001 and 0.01, respectively.

Drought stress is one of the important environmental stresses and has adverse impacts on the crop productivity. Foliar application of fertilizers could enhance the use efficiency of a nutrient urgently needed for the growth and development of plant^38–40^. Urea is a widely utilized foliar N fertilizer, with characteristics of high leaf penetration rate and low cost^41^. Foliar application of nitrogen was sometimes carried when abiotic stress occurred^22^. It has been reported that improving plant nutrition by increasing the supply of nutrients, including nitrogen may ameliorate the negative effects of abiotic stress^42,43^. Supplemental foliar application of urea could effectively enhance the growth, yield and yield components of sunflower subjected to drought stress^44^. These results were in agreement with the work of Zhang et al.^22^, who reported that foliar application of nitrogen posed positive impacts on the growth of maize under short-term drought stress. The previous study demonstrated that foliar application of α-oxoglutarate significantly increased the fresh weight of soybean seedling exposed to cold stress^33^. Similarly, the present study indicated that foliar nitrogen, foliar α-oxoglutarate and a combination of foliar nitrogen plus α-oxoglutarate resulted in a marked rise in the fresh weight of soybean seedling exposed to drought stress as compared to CK1 at S1, S2, and S3 stages.

### Effects of foliar α-oxoglutarate and nitrogen on relative water content

Leaf RWC is reported to be an alternative measure of evaluating plant water status. As shown in Table 2, foliar application of nitrogen and α-oxoglutarate significantly increased RWC of soybean leaf under drought stress compared to CK1 at S1, S2 and S3 stages. Leaf RWC under DS1, DS2, and DS3 was significantly greater than that under CK2 at each stage. There was no significant difference in RWC between DS1 and DS2 treatments and the RWC under DS3 was significantly higher than that under DS1 and DS2 at S1 and S2 stages. The leaf RWC under drought stress gradually decreased with the stress duration prolonged. The leaf RWC under DS1, DS2, DS3, and CK1 gradually decreased from S1 to S3 stage. The RWC of Hefeng51 was significantly greater than that for Hefeng43 at each stage. The data as shown in Table 2 showed that there was a lower reduction in RWC for Hefeng51 than that for Hefeng43 from S1 to S3 stage when subjected to drought stress. The RWC was significantly affected by treatment (T), cultivar (C) and their interaction (C × T) at each stage.

**Table 2:** Effects of foliar α-oxoglutarate and nitrogen on leaf RWC under drought stress (%).

Treatment	S1 stage		S2 stage		S3 stage	
	Hefeng51	Hefeng43	Hefeng51	Hefeng43	Hefeng51	Hefeng43
DS1	58.24^c^	53.66^c^	55.25^c^	50.09^c^	52.25^b^	46.25^b^
DS2	59.36^c^	53.81^c^	56.18^c^	50.21^c^	53.02^b^	46.65^b^
DS3	63.85^b^	59.95^b^	61.27^b^	54.16^b^	53.56^b^	47.34^b^
CK1	54.67^d^	50.52^d^	52.92^d^	46.67^d^	45.25^c^	38.43^c^
CK2	68.21^a^	63.21^a^	67.98^a^	64.12^a^	68.46^a^	63.56^a^
**Analysis of variance (ANOVA)**						
Treatment (T)	*	**	**	***	***	***
Cultivar (C)	**		**		***	
C × T	*		**		***	

Means with same letter within columns are not significantly different (*P* < 0.05) using LSD test.

***, **, *Significance at 0.001, 0.01, and 0.05, respectively.

It is important for plants to keep desirable RWC, which relieves the adverse impacts of drought stress^5,45,46^. This study is similar to the work of Taiz et al.^5^ who reported that the RWC was greater for plants under control conditions than those under drought stress. It has been proved that foliar application of nitrogen could cause a marked rise in RWC of both maize varieties exposed to drought stress^22^, which is similar to the results obtained in the present study. No information is available on the effects of a combination of foliar nitrogen plus α-oxoglutarate on the RWC of plant under abiotic stress. The data in this study showed that foliar nitrogen significantly enhanced the leaf RWC of soybean compared to CK1 at each stage, and the combination of foliar nitrogen plus α-oxoglutarate showed a much more positive effect than that under foliar nitrogen or α-oxoglutarate at S1 and S2 stages, while there is no significant difference in leaf RWC among DS1, DS2, and DS3 treatments at S3 stage.

### Effects of foliar α-oxoglutarate and nitrogen on proline content

Proline accumulation is found to play adaptive roles in stress resistance^47^. The proline content was significantly influenced by treatment (T), cultivar (C) and their interaction (C × T) at each stage (Table 3). The proline content of Hefeng51 was significantly greater than that for Hefeng43 at each stage. The data in Table 3 showed that there was higher proline content for Hefeng51 than that for Hefeng43 at each stage when exposed to drought stress. That suggested that Hefeng51 could better adapt to drought stress than Hefeng43 in the present study. Similar to the work conducted by You et al.^47^ who found that the drought-tolerant sesame accumulates higher levels of proline, lysine, arginine, and allantoin under drought conditions.

**Table 3:** Effects of foliar α-oxoglutarate and nitrogen on leaf proline content under drought stress (µg g^−1^ FW).

Treatment	S1 stage		S2 stage		S3 stage	
	Hefeng51	Hefeng43	Hefeng51	Hefeng43	Hefeng51	Hefeng43
DS1	60.51^b^	56.09^b^	75.99^b^	71.12^b^	86.93^b^	79.23^b^
DS2	61.80^b^	57.15^b^	74.42^b^	72.04^b^	86.77^b^	78.86^b^
DS3	70.30^a^	64.67^a^	85.95^a^	83.94^a^	92.85^a^	87.22^a^
CK1	50.36^c^	43.86^c^	68.28^c^	58.04^c^	77.12^c^	64.67^c^
CK2	35.91^d^	31.97^d^	35.23^d^	32.06^d^	36.58^d^	31.67^d^
Treatment (T)	***	***	***	***	***	***
Cultivar (C)	***		***		***	
C × T	**		***		***	

Means with same letter within columns are not significantly different (*P* < 0.05) using LSD test.

***, **Significance at 0.001, and 0.01, respectively.

Results obtained from the present study demonstrated that foliar application of nitrogen and α-oxoglutarate significantly enhanced leaf proline content of soybean under drought stress compared to CK1 at S1, S2, and S3 stages (Table 3). Leaf proline content of soybean under DS1, DS2, and DS3 was significantly higher than that under CK2 at each stage. There was no significant difference in leaf proline content between DS1 and DS2 treatments at each stage, while the leaf proline content under DS3 was significantly greater than that under DS1 and DS2 at S1 and S2 stages. The leaf proline content under drought stress gradually increased with the stress duration prolonged. This could be greatly vital for the crop to adapt to drought stress^48^. In addition, lettuce plants under salt stress were sprayed with urea, and the contents of glycine and proline increased further^49^. N nutrition can alleviate crop water stress by maintaining metabolic activities. Under deficit irrigation, nitrogen treated plants had higher growth and yield characteristics. Compared with the plants without nitrogen treatment, free proline was significantly improved due to nitrogen application, which effectively reduced the damage of wheat plants under drought stress^50^.

The role of proline in drought resistance has been investigated and positive associations between improved drought tolerance and proline accumulation have been found in the published report^51^. To date, there have been no reports on the effects of a combination of foliar nitrogen plus α-oxoglutarate on the regulation of proline accumulation in crops. However, in increasing the proline accumulation under drought stress, a combination of foliar nitrogen plus α-oxoglutarate had an advantage over either foliar nitrogen or foliar α-oxoglutarate in the present study (Table 3). It is important to take preventive actions to alleviate the adverse effects of drought stress. The data demonstrated that foliar application of nitrogen and α-oxoglutarate significantly increased leaf proline content at each stage. These findings supported several studies that have provided the experimental evidence of the role of nitrogen in ameliorating the adverse impacts of drought stress by enhancing proline accumulation and soluble protein^18–21^. Li et al.^28^ pointed out that foliar application of exogenous α-oxoglutarate could enhance the proline content in wheat leaf and mitigate the adverse impacts of drought stress on wheat growth and development. Their results revealed the importance of exogenous α-oxoglutarate for wheat exposed to drought stress.

### Effects of foliar α-oxoglutarate and nitrogen on glutamate content

As shown in Table 4, the glutamate content was significantly affected by treatment (T), cultivar (C) and their interaction (C × T) at each stage. The glutamate content of Hefeng51 was significantly higher than that for Hefeng43 at each stage. The data obtained in the present study demonstrated that there was a lower reduction in glutamate content for Hefeng51 than that for Hefeng43 from S1 to S3 stage when exposed to drought stress. Data from the present study indicated that foliar application of nitrogen and α-oxoglutarate caused a significant rise in leaf glutamate content of soybean seedling under drought stress as compared with CK1 at each stage (Table 4). Leaf glutamate content of soybean seedling under DS1, DS2, and DS3 was significantly greater than that under CK2 at S1, S2, and S3 stages. There was no significant difference in leaf glutamate content between DS1 and DS2 treatments at each stage, while the leaf glutamate content under DS3 was significantly greater than that under DS1 and DS2 at S1 and S2 stages. The leaf glutamate content under DS1, DS2, DS3, and CK1 gradually decreased from S1 to S3 stage.

**Table 4:** Effects of foliar α-oxoglutarate and nitrogen on leaf glutamate content under drought stress (µg g^−1^ FW).

Treatment	S1 stage		S2 stage		S3 stage	
	Hefeng51	Hefeng43	Hefeng51	Hefeng43	Hefeng51	Hefeng43
DS1	189.08^b^	175.32^b^	179.04^b^	163.79^b^	165.88^a^	139.25^a^
DS2	191.53^b^	176.01^b^	181.65^b^	164.38^b^	166.15^a^	140.58^a^
DS3	211.25^a^	198.36^a^	204.78^a^	178.27^a^	170.86^a^	141.89^a^
CK1	175.85^c^	154.25^c^	164.45^c^	140.24^c^	150.03^b^	112.21^b^
CK2	116.91^d^	105.21^d^	117.34^d^	104.69^d^	116.25^c^	103.28^c^
**Analysis of variance (ANOVA)**						
Treatment (T)	***	***	***	**	**	*
Cultivar (C)	***		**		**	
C × T	***		**		**	

Means with same letter within columns are not significantly different (*P* < 0.05) using LSD test.

***, **, *Significance at 0.001, 0.01, and 0.05, respectively.

An adequate supply of glutamate (Glu) is required for proline synthesis rate^46^. Similar to the present study, foliar application of α-oxoglutarate for plant had a positive impact on the rise of glutamate content^33,52^. This study also suggested foliar application of nitrogen plus α-oxoglutarate much more significantly enhanced leaf glutamate content under drought stress than that at DS1 or DS2 at S1 and S2 stages (Table 4).

### Effects of foliar α-oxoglutarate and nitrogen on GS and GDH activity

The data in Tables 5 and 6 indicated there was no significant difference in leaf GS and GDH between DS1 and DS2 treatments at each stage, while the leaf activity of GS and GDH under DS3 was significantly greater than that under DS1 and DS2 at S1 and S2 stages. With the stress duration prolonged, the leaf activity of GS and GDH gradually decreased under drought stress. Drought stress resulted in a significant decrease in GS activity compared to CK2 at each stage, while drought stress led to a marked rise in GDH activity as compared with CK2 at S1, S2, and S3 stages. The data obtained in this study showed that foliar application of nitrogen and α-oxoglutarate significantly enhanced the leaf activity of GS and GDH of soybean seedling under drought stress as compared with CK1 at each stage (Tables 5, 6). The leaf activity of GS and GDH under DS1, DS2, and DS3 treatments with foliar nitrogen and α-oxoglutarate exhibited a lower reduction than that under CK1 from S1 to S3 stage.

**Table 5:** Effects of foliar α-oxoglutarate and nitrogen on leaf GS activity under drought stress (µmol h^−1^ g^−1^ FW).

Treatment	S1 stage		S2 stage		S3 stage	
	Hefeng51	Hefeng43	Hefeng51	Hefeng43	Hefeng51	Hefeng43
DS1	180.98^c^	151.25^c^	171.25^c^	140.33^b^	152.32^b^	120.03^b^
DS2	183.26^c^	153.02^c^	172.37^c^	141.19^b^	155.18^b^	121.65^b^
DS3	191.95^b^	171.43^b^	182.49^b^	155.83^b^	158.87^b^	123.29^b^
CK1	165.47^d^	142.35^d^	148.46^d^	122.34^c^	136.14^c^	102.87^c^
CK2	218.13^a^	182.67^a^	219.81^a^	184.11^a^	220.54^a^	185.05^a^
**Analysis of variance (ANOVA)**						
Treatment (T)	***	***	***	***	***	**
Cultivar (C)	***		***		**	
C × T	***		**		**	

Means with same letter within columns are not significantly different (*P* < 0.05) using LSD test.

***, **Significance at 0.001, and 0.01, respectively.

**Table 6:** Effects of foliar α-oxoglutarate and nitrogen on GDH activity under drought stress (µmol h^−1^ g^−1^ FW).

Treatment	S1 stage		S2 stage		S3 stage	
	Hefeng51	Hefeng43	Hefeng51	Hefeng43	Hefeng51	Hefeng43
DS1	19.26^b^	15.96^b^	18.18^b^	13.18^b^	16.95^a^	11.05^a^
DS2	19.05^b^	16.01^b^	18.21^b^	13.21^b^	16.91^a^	11.11^a^
DS3	20.87^a^	16.92^a^	19.25^a^	14.25^a^	17.06^a^	11.26^a^
CK1	16.23^c^	13.65^c^	15.02^c^	11.92^c^	13.15^b^	9.15^b^
CK2	11.21^d^	9.18^d^	11.12^d^	9.12^d^	11.31^c^	9.07^b^
**Analysis of variance (ANOVA)**						
Treatment (T)	***	***	**	***	**	*
Cultivar (C)	**		***		**	
C × T	**		**		*	

Means with same letter within columns are not significantly different (*P* < 0.05) using LSD test.

***, **, *Significance at 0.001, 0.01, and 0.05, respectively.

Little research has been done on the effects of nitrogen and α-oxoglutarate on leaf GS and GDH activity of soybean exposed to drought stress. An early published report demonstrated that the GS/GOGAT pathway was reported to play a key role in glutamate synthesis when proline is required^53^. There were some studies indicating that the increase in NADH-GDH activity and its vital role in providing glutamate for proline biosynthesis^54,55^. Luo et al.^56^ pointed out that foliar application of α-oxoglutarate could effectively enhance the leaf activity of GDH and GS in wheat when exposed to drought stress. The data in the present study clearly demonstrated that foliar α-oxoglutarate, foliar nitrogen and a combination of foliar nitrogen plus α-oxoglutarate imposed positive effects on the rise of GS and GDH activity for soybean seedling subjected to drought stress (Tables 5, 6). This finding was also similar to the work conducted by Zhang et al.^22^ that foliar application of nitrogen was beneficial to plant growth and the increase of nitrogen metabolism in both maize varieties when subjected to short-term drought stress. In the present study, a rise in the activity of GS and GDH caused by foliar nitrogen and α-oxoglutarate could explain the reason why ammonium content in soybean leaf was accordingly reduced when soybean seedlings were exposed to drought stress (Tables 5, 6, 7). The effect of foliar nitrogen and α-oxoglutarate on nitrogen assimilation was also reflected by a markedly lower level of ammonium in the present study (Table 7).

**Table 7:** Effects of foliar α-oxoglutarate and nitrogen on ammonium content under drought stress (µmol h^−1^ g^−1^ FW).

Treatment	S1 stage		S2 stage		S3 stage	
	Hefeng51	Hefeng43	Hefeng51	Hefeng43	Hefeng51	Hefeng43
DS1	1.27^c^	1.85^c^	1.37^b^	2.11^b^	1.58^b^	2.45^b^
DS2	1.22^c^	1.81^c^	1.35^b^	2.08^b^	1.60^b^	2.51^b^
DS3	1.01^b^	1.54^b^	1.19^c^	1.81^b^	1.69^b^	2.60^b^
CK1	1.52^a^	2.38^a^	1.78^a^	2.87^a^	2.04^a^	3.27^a^
CK2	0.65^d^	0.68^d^	0.63^d^	0.71^c^	0.64^c^	0.67^c^
**Analysis of variance (ANOVA)**						
Treatment (T)	*	**	**	***	***	***
Cultivar (C)	***		***		***	
C × T	**		**		***	

Means with same letter within columns are not significantly different (*P* < 0.05) using LSD test.

***, **, *Significance at 0.001, 0.01, and 0.05, respectively.

### Effects of foliar α-oxoglutarate and nitrogen on ammonium content

The data in Table 7 demonstrated that leaf ammonium content was significantly reduced by foliar application of α-oxoglutarate and nitrogen at each stage. There was a lower increment in leaf ammonium content after exposure to foliar α-oxoglutarate and nitrogen at DS1, DS2 and DS3 than that at CK1 from S1 to S3 stage. The proline ammonium content was significantly influenced by treatment (T), cultivar (C) and their interaction (C × T) at each stage (Table 7). The ammonium content in Hefeng51 was significantly lower compared to Hefeng43 when exposed to drought stress at each stage. The data in Table 7 showed that there was a lower increment in ammonium content for Hefeng51 than that for Hefeng43 from S1 to S3 stage when exposed to drought stress.

Excessive ammonium could pose toxic effects on crops^11^. The GDH pathway is considered as a complementary route when plants are exposed to abiotic stresses^12,33^. The data in the present study indicated that foliar nitrogen and α-oxoglutarate enhanced GS activity, GDH activity, and resulted in the reduction in ammonium content in soybean leaf. Similarly, an early report showed that foliar application of α-oxoglutarate could promote the ammonium assimilation in roots and shoots of tomato, suggesting that the availability of carbon skeletons is a key limiting factor for ammonium uptake in tomato plants^32^. Yuan et al. also observed the increase in GS activity and the ammonium uptake when exogenous α-oxoglutarate was applied to rice exposed to abiotic stress^31^. The data in the present study indicated that foliar α-oxoglutarate significantly increased proline content and reduced ammonium content of soybean seedlings exposed to drought stress. Moreover, a combination of foliar α-oxoglutarate plus nitrogen mitigated the adverse effects of drought stress on soybean seedling better than foliar α-oxoglutarate or nitrogen at S1 and S2 stages. In the end, an increase in proline content and RWC could explain the reason why foliar nitrogen and α-oxoglutarate could alleviate the adverse impacts of drought stress on soybean seedlings.

### Effects of foliar α-oxoglutarate and nitrogen on chlorophyll content (Ch) and photosynthetic rate (Pn)

Since photosynthesis is closely related to plant growth, photosynthesis-related parameters such as photosynthetic rate and chlorophyll content were determined in the present study. The data obtained in the present study indicated that the chlorophyll content and photosynthetic rate were significantly affected by foliar α-oxoglutarate and nitrogen (Tables 8, 9). Results from this study showed that there was no significant difference in leaf chlorophyll content and photosynthetic rate between DS1 and DS2 treatments at each stage, while the leaf chlorophyll content and photosynthetic rate at DS3 were significantly greater than that at DS1 and DS2 at S1 and S2 stages. The leaf chlorophyll content and photosynthetic rate under drought stress gradually decreased with the stress duration prolonged. The data obtained in this study showed that foliar application of nitrogen and α-oxoglutarate significantly enhanced the leaf chlorophyll content and photosynthetic rate of soybean seedling under drought stress compared to CK1 at each stage. The leaf chlorophyll content and photosynthetic rate under DS1, DS2, and DS3 treatments with foliar nitrogen and α-oxoglutarate resulted in a lower reduction than that under CK1 from S1 to S3 stage. A combination of foliar nitrogen plus α-oxoglutarate was better in improving photosynthesis under drought stress than either foliar nitrogen or foliar α-oxoglutarate.

**Table 8:** Effects of foliar α-oxoglutarate and nitrogen on leaf relative chlorophyll content under drought stress (*SPAD*).

Treatment	S1 stage		S2 stage		S3 stage	
	Hefeng51	Hefeng43	Hefeng51	Hefeng43	Hefeng51	Hefeng43
DS1	46.25^c^	43.06^c^	44.25^c^	40.85^c^	42.57^b^	37.35^b^
DS2	46.49^c^	43.19^c^	44.49^c^	40.97^c^	42.69^b^	37.62^b^
DS3	48.36^b^	45.08^b^	46.46^b^	41.28^b^	43.26^b^	37.78^b^
CK1	43.05^d^	37.17^d^	41.55^d^	36.37^d^	38.21^c^	33.01^c^
CK2	52.26^a^	48.57^a^	52.69^a^	49.07^a^	53.19^a^	49.43^a^
**Analysis of variance (ANOVA)**						
Treatment (T)	*	**	*	**	**	***
Cultivar (C)	**		**		***	
C × T	*		**		**	

Means with same letter within columns are not significantly different (*P* < 0.05) using LSD test.

***, **, *Significance at 0.001, 0.01, and 0.05, respectively.

**Table 9:** Effects of foliar α-oxoglutarate and nitrogen on photosynthetic rate under drought stress (µmol CO_2_ m^−2^ s^−1^).

Treatment	S1 stage		S2 stage		S3 stage	
	Hefeng51	Hefeng43	Hefeng51	Hefeng43	Hefeng51	Hefeng43
DS1	23.79^b^	20.89^c^	22.09^c^	18.54^c^	20.67^c^	16.38^c^
DS2	24.08^b^	21.06^c^	22.18^c^	18.86^c^	20.71^c^	16.25^c^
DS3	25.75^b^	23.51^b^	24.36^b^	20.31^b^	22.94^b^	18.06^b^
CK1	21.85^c^	19.87^d^	19.84^d^	16.25^d^	17.80^d^	14.01^d^
CK2	28.28^a^	22.12^a^	28.31^a^	22.38^a^	26.48^a^	22.38^a^
**Analysis of variance (ANOVA)**						
Treatment (T)	*	*	*	**	**	***
Cultivar (C)	*		**		**	
C × T	*		*		**	

Means with same letter within columns are not significantly different (*P* < 0.05) using LSD test.

***, **, *Significance at 0.001, 0.01, and 0.05, respectively.

The photosynthetic rate and chlorophyll content (*SPAD*) are two key parameters of photosynthesis. It has been observed in previous reports demonstrating that foliar application of α-oxoglutarate posed positive influence on leaf photosynthesis^57–60^. The data in this study was in agreement with the study by Ge^59^ who pointed out that foliar application of α-oxoglutarate enhanced the leaf photosynthetic rate and chlorophyll content of winter-wheat when subjected to drought stress. In the present study, foliar application of α-oxoglutarate and nitrogen enhanced the nitrogen metabolism, assimilated more ammonium and thus resulted in the increase of proline content at S1, S2, and S3 stages. NADPH is required when proline biosynthesis takes place and NADP^+^ which is needed for photosynthesis is derived from NADPH decomposition^61^. Based on the results above, it was found that exogenous α-oxoglutarate and nitrogen might enhance photosynthetic rate in leaf through increasing the proline content of soybean seedling under drought stress.

### Analysis of correlation between physio-chemical traits under drought stress

**Figure 1:**
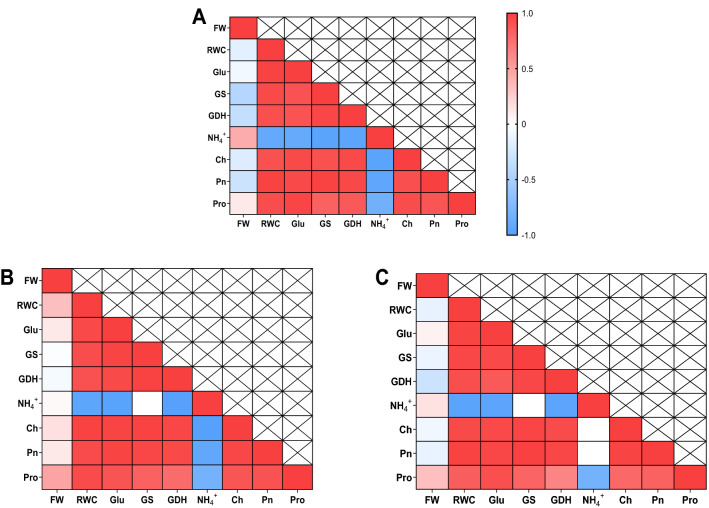
The correlation between physio-chemical traits of soybean leaf at S1 (**A**), S2 (**B**), and S3 (**C**) stages under drought stress.

Analysis of correlation between all parameters under drought stress was performed to understand the relationship among all variables in soybean seedling exposed to drought stress with the application of foliar α-oxoglutarate and nitrogen. As shown in Fig. 1, all parameters exhibited statistically positive correlations with proline except for ammonium (NH_4_^+^) at each stage. The finding in this study was consistent with the study by Ren et al.^52^ who confirmed the role of proline in drought resistance and found the positive correlations between proline accumulation and improved drought tolerance. There has been a report that glutamate biosynthesis is greatly linked with ammonium assimilation^10^. The data obtained in this study suggested that there was positively significant relationship between glutamate and proline, which were negatively correlated with ammonium (NH_4_^+^) at each stage. Positive correlation between glutamate and proline was observed at S1, S2, and S3 stages with a correlation coefficient of 0.971**, 0.919**, and 0.893**, respectively, which supported glutamate is required when the rate of proline biosynthesis is enhanced^47^. Under drought stress, GDH activity showed positively significant relationship with glutamate but negatively significant relationship with ammonium (NH_4_^+^), indicating that GDH pathway could be considered as a complementary role in assimilating ammonium under abiotic stress^13–15^. NADPH is required when proline biosynthesis take place and NADP^+^ which is needed for photosynthesis, is derived from NADPH decomposition. Similarly, this study indicated that proline showed statistically positive relationship with Pn and Ch at each stage. This revealed that an increase in proline content was beneficial to photosynthesis under drought stress.

## Conclusions

The present study has added new information that foliar α-oxoglutarate and nitrogen improved the drought tolerance in two soybean cultivars. Foliar α-oxoglutarate and nitrogen significantly increased leaf GS activity, GDH activity, glutamate content, proline content and photosynthesis of soybean seedlings exposed to drought stress at each stage. Accordingly, the ammonium content was significantly reduced by foliar α-oxoglutarate and nitrogen. These results suggested that in increasing the proline accumulation under drought stress, a combination of foliar nitrogen plus α-oxoglutarate had an advantage over either foliar nitrogen or foliar α-oxoglutarate, and a combination of foliar nitrogen plus α-oxoglutarate could mitigate the adverse effects of drought stress.

## Data Availability

All data are contained in this article.
